# The Cyclic Antimicrobial Peptide C-LR18 Has Enhanced Antibacterial Activity, Improved Stability, and a Longer Half-Life Compared to the Original Peptide

**DOI:** 10.3390/antibiotics14030312

**Published:** 2025-03-17

**Authors:** Zhihua Pei, Qiaoxi Song, Jingqi Xu, Shuang Yu, Hongxia Ma

**Affiliations:** 1 College of Veterinary Medicine, Jilin Agricultural University, Xincheng Street No. 2888, Changchun 130118, China; songqiaoxi@mails.jlau.edu.cn (Q.S.); jingqixu@mails.jlau.edu.cn (J.X.); ys18840107955@163.com (S.Y.); 2 Key Laboratory of New Veterinary Drug Research and Development of Jilin Province, Xincheng Street No. 2888, Changchun 130118, China

**Keywords:** cyclic antimicrobial peptide, cyclization, stability, half-life

## Abstract

**Background:** LR18 is an α₋helical AMP with high antimicrobial activity, low hemolytic activity, and low cytotoxicity. However, the susceptibility to degradation of the peptidase enzyme and a short half-life hinder its application as a therapeutic agent. Improving the stability and prolonging the half-life of LR18 are crucial to accelerate its application in the treatment of infectious diseases. **Methods:** A new cyclic peptide, C-LR18, was designed and synthesized through end-to-end cyclization of LR18 via disulfide bonds. The biological activity, half-life, and therapeutic effect of C-LR18 on *Escherichia coli*₋infected mice were studied. **Results:** C-LR18 maintained the characteristics of low cytotoxicity and low hemolytic activity of the original LR18 peptide and had higher antibacterial activity and significantly improved stability. After treatment with 1 mg/mL of trypsin, carboxypeptidase, and papain for 1 h, the MIC of C-LR18 against *E. coli* ATCC25922 was 4 μM, while that of LR18 had increased to 128 μM. After exposure to 50% serum or artificial gut solution for 30 min, the MIC of C-LR18 against *E. coli* ATCC25922 increased 4-fold, while that of LR18 increased 16-fold. The half-life of C-LR18 in plasma and in rats was extended to 3.37-fold and 4.46-fold, respectively, that of LR18. The acute toxicity of C-LR18 in mice is lower than many AMPs reported so far (LD50 = 37.8 mg/kg). C-LR18 has a therapeutic effect on *E.coli*-infected mice. **Conclusions:** The cyclic peptide C-LR18 has higher antibacterial activity and stability and a longer half-life than LR18 in rats in vitro and in vivo. C-LR18 also has a therapeutic effect on KM mice infected with *E. coli* and is expected to become a therapeutic drug for bacterial diseases and applied to the treatment of human and veterinary diseases.

## 1. Introduction

With the increasing resistance of pathogens to common antibiotics, the development of new antimicrobial drugs and their analogues has become an urgent need for current research in the treatment of human and animal diseases. Antimicrobial peptides (AMPs) with unique antimicrobial mechanisms are currently attracting increasing interest as therapeutic candidates. However, most AMPs have the disadvantages of low oral bioavailability, high risk of proteolytic degradation, and high excretion rate, which greatly influence their development in therapeutic fields, especially when administered intravenously or orally [1,2,3,4]. Therefore, improving the stability and prolonging the in vitro and in vivo half-life of AMPs have become the focus of research and development of new AMP drugs.

There are many methods to improve the stability and prolong the half-life of peptides in the literature, including chemical modification (cyclization [5], halogenation [6], terminal modification [7,8,9], etc.), unnatural amino acid modification [10,11,12], and the design of self-assembled nanomaterials [13,14]. Cyclization of a peptide refers to a modification method that uses chemical methods to cyclize the head and tail of a linear peptide or partially cyclize it to make it into a more stable ring structure and extend its half-life. Compared with linear peptides, cyclic peptides have stronger biological activity, protease (including exopeptidase and endopeptidase) stability, and cell selectivity [15,16,17,18]. Therefore, cyclization of linear AMPs is expected to be an effective method to improve the stability of AMPs, prolong the half-life of AMPs, and improve their absorption and utilization in vivo.

Cyclization of peptides can be achieved chemically via techniques such as head to side chain, side chain to side chain, head to tail, and side chain to tail, which are determined by the functional groups [16,19,20,21]. LR18 is an α-helical AMP designed by our research team that is mainly composed of leucine and arginine. Our research results have shown that LR18 has high antimicrobial activity, low hemolytic activity, and low cytotoxicity [22]. However, the susceptibility to degradation of the peptidase enzyme and a short half-life hinder its application as a therapeutic agent. In order to improve the antibacterial activity and stability of the antimicrobial peptide LR18, prolong its half-life, and promote its clinical application, in this study, cysteine was added to the N-terminal and C-terminal of the linear peptide LR18, and a new cyclic antimicrobial peptide, named C-LR18, was synthesized by means of end-to-end cyclization via disulfide bonds. The biological activity, half-life, and therapeutic effect of C-LR18 on *E. coli*-infected mouse models were studied. This provides a basis for the study of obtaining new antimicrobial drugs via the structural modification of existing antimicrobial peptides.

## 2. Results

### 2.1. Design and Physicochemical Parameters of Cyclic Peptide C-LR18

**Figure 1:**
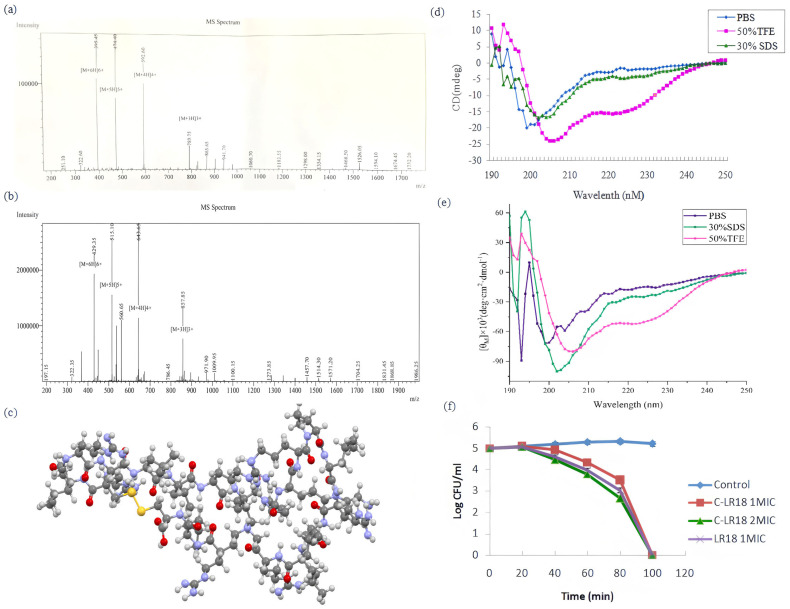
The LC-MS spectrum, circular dichroism (CD) spectra, 3D structure diagram of C-LR18, and its bactericidal kinetic curves on *E. coli* ATCC25922. (**a**) The LC-MS spectrum of LR18; (**b**) the LC-MS spectrum of C-LR18; (**c**) the 3D structure diagram of C-LR18; (**d**) the circular dichroism (CD) spectra of LR18 in 10 mM PBS, 50% TFE, and 30% SDS; (**e**) the circular dichroism (CD) spectra of C-LR18 in 10 mM PBS, 50% TFE, and 30% SDS; (**f**) the bactericidal kinetic curves of C-LR18 on *E. coli* ATCC25922.

In this study, cysteine was added to the N-terminal and C-terminal of the linear peptide LR18, and a new cyclic peptide with a stable stereostructure named C-LR18 was synthesized by means of end-to-end cyclization via disulfide bonds. The LC-MS spectrum of C-LR18 is shown in Figure 1b. The sequences, molecular weights, and isoelectric points of C-LR18 and LR18 are shown in Table 1. Biological software (http://web.expasy.org/ (accessed on 21 March 2021)) analysis showed that the static charge of the cyclic peptide C-LR18 was the same as that of the original peptide LR18, which was +8, the instability index of C-LR18 was less than LR18, and the average hydrophobicity of C-LR18 was also less than LR18.

### 2.2. The Cyclic Peptide C-LR18 Still Has α-Helical Structure

The secondary structure of antimicrobial peptide C-LR18 in 10 mM PBS, 50% TFE, and 30 mM SDS was detected via circular dichroism. As shown in Figure 1e, C-LR18 showed a disordered conformation in 10 mM PBS. In 30 mM SDS, the environment of simulated negatively charged nuclear film, there is a tendency to form α-helical structure with an obvious negative peak at 202 nm. In 50% TFE, the hydrophobic environment of simulated biofilm, the first negative peak is at 205 nm, and the second negative peak is at 222 nm, showing a typical alpha-helical structure. The results showed that the peptide sequence still had the αhelical structure after cyclization of LR18. Figure 1c shows the 3D structure diagram of C-LR18. It has a ring structure as a whole, and the yellow part indicates the position of disulfide bonds.

### 2.3. The Antibacterial Activity of Cyclic Peptide C-LR18 Was Enhanced

In this study, the geometric mean MIC (MBC) values of C-LR18 and LR18 against the tested strains were calculated so as to intuitively reflect the average antibacterial effect of the two peptides. As shown in Table 2, C-LR18 had higher antibacterial activity against both Gram-positive and Gram-negative bacteria than linear peptide LR18. The inhibitory activity against Gram-negative bacteria [Geometric mean (GM) MIC = 2 μM] was significantly higher than that against Gram-positive bacteria (GM MIC = 8.7 μM). The bactericidal kinetic effects of C-LR18 on *E. coli* ATCC25922 were also investigated. The results shown in Figure 1f indicate that *E. coli* ATCC25922 could be completely killed by C-LR18 within 100 min in a dose-dependent manner.

### 2.4. The Antibacterial Mechanism Study

**Figure 2:**
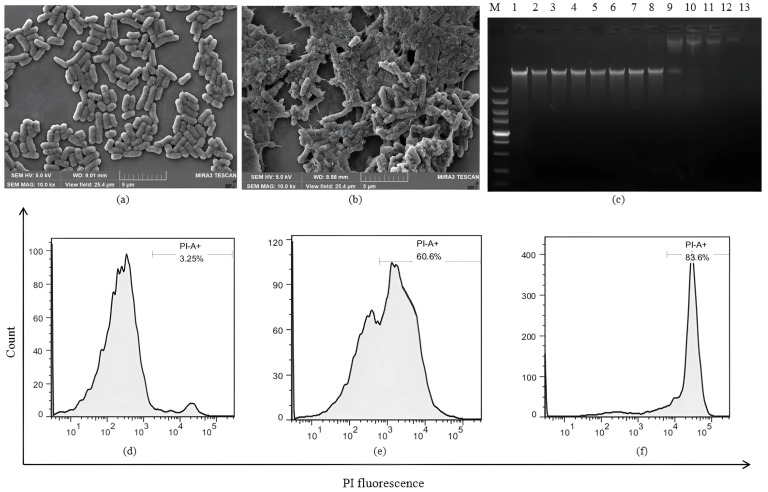
The antimicrobial mechanism of C-LR18 against *E. coli* ATCC25922. (**a**) Bacterial morphology of *E. coli* ATCC25922 treated with PBS (control) under scanning electron microscope; (**b**) bacterial morphology of *E. coli* ATCC25922 treated with C-LR18 under scanning electron microscope; (**c**) DNA binding assay results of C-LR18. M: DNA Marker; Lane 1: genomic DNA of *E. coli* ATCC25922; Lane 2–12: the mixture of *E. coli* ATCC25922 genomic DNA and LR18 with the concentrations of 0.5, 1, 2, 4, 8, 16, 32, 64, 128, 256, and 512 µM; Lane13: only C-LR18; (**d**) *E. coli* ATCC25922 treated with PBS (negative control); (**e**) *E. coli* ATCC25922 treated with 1 × MIC C-LR18; (**f**) *E. coli* ATCC25922 treated with melittin (4 μM) (positive control).

The cell morphology and membrane damage of *E. coli* ATCC25922 treated with C-LR18 were observed by SEM. Compared to the control group, *E. coli* treated with C-LR18 at 1 × MIC for 0.5 h appeared a rough surface with blebbing, as well as shrunken and destroyed shapes, multiple bacteria condense into clumps (Figure 2b). The membrane permeability ability of C-LR18 on *E. coli* ATCC25922 was detected. Compared with the negative control (treated with PBS), the fluorescence intensity of *E. coli* ATCC25922 cells treated with C-LR18 increased by 60.6% (*p* < 0.01) (Figure 2e). The results showed that C-LR18 increased the permeability of the bacterial cell membrane, allowing PI to enter the cells and embed into DNA, and the membrane permeability was higher than LR18 [22]. The DNA binding analysis showed that C-LR18 exhibited DNA-binding ability at 64 µM (Figure 2c), which had the same DNA-binding ability as LR18 [22]. Because the DNA-binding concentration of C-LR18 is much higher than its MICs, C-LR18 exerts its bactericidal effect mainly by acting on the cell membrane.

### 2.5. The Hemolytic Activity and Cytotoxicity Determination

**Figure 3:**
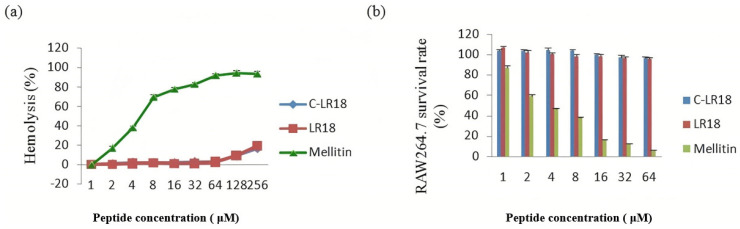
Results of hemolytic activity and cytotoxicity assay of peptide C-LR18. (**a**) Hemolytic activity of peptides on rabbit red blood cells; (**b**) cytotoxicity of peptides on RAW264.7 cells. The graphs are based on at least three independent experiments.

In this study, the hemolytic activity of cyclic peptide C-LR18 on rabbit erythrocytes was detected. The minimum hemolysis concentration of 10% (MHC10) was characterized as the lowest concentration inducing 10% hemolysis, and the results are shown in Table 2 and Figure 3a. When the concentration of C-LR18 was less than or equal to 64 μg/mL, the hemolytic rate was less than 10%, which was similar to that of LR18, but significantly lower than that of melittin. The results indicated that the hemolytic activity of the modified peptide did not increase, and the advantage of the low hemolyticity of the original peptide was retained. In this study, the therapeutic index (TI) (MHC10/GMMIC) of C-LR18 was also calculated (Table 2). The GMTI of cyclic peptide C-LR18 (14.71/64) against both Gram-positive and Gram-negative bacteria was higher than LR18 (13.76/47.4).

The cytotoxicity test results of peptides C-LR18 and LR18 on mouse mononuclear macrophage *RAW264.7* cells are shown in Figure 3b, and the cytotoxicity is significantly lower than that of melitin. When the concentrations of antimicrobial peptides C-LR18 and LR18 were less than or equal to 64 μM, there was little effect on the survival rate of eukaryotic cells, indicating that C-LR18 retained the advantage of low cytotoxicity of the propeptide after cyclization, which was consistent with the results of hemolytic activity.

### 2.6. The Stability of Cyclic Peptide C-LR18 Was Significantly Improved

The stability of LR18 and C-LR18 in protease, serum, and Gastrointestinal Conditions was determined using the MIC assay. According to the results shown in Table 3, cyclic peptide C-LR18 still maintained high antibacterial activity against *E. coli* after being treated with trypsin, carboxypeptidase, and papain for 1h, and its MIC was 4/8 μM, while the inhibitory activity of linear peptide LR18 against *E. coli* was decreased to different degrees. The results indicated that the protease stability of the cyclic peptide was significantly improved. In this study, the antibacterial activity of C-LR18 and LR18 against *E. coli* ATCC25922 was measured in rat serum and fetal bovine serum at different concentrations. As shown in Table 3, the antibacterial activity of the two peptides decreased with the increase in the serum concentration and was correlated with the concentration. When the serum concentration reached 50%, the MIC value of C-LR18 only increased by 4 times, while the MIC of original peptide LR18 increased by 16 times, indicating that the serum stability of antimicrobial peptides after cyclized modification was improved to a certain extent. In this study, the MIC of C-LR18 against *E. coli* ATCC25922 was determined after treatment with artificial gastric juice and artificial intestinal juice. As shown in Table 3, the MIC of C-LR18 increased by 4 times and 16 times after treatment with artificial intestinal fluid for 30 min and 60 min, respectively, while the MIC of LR18 increased by 8 times and 32 times, respectively, after treatment with artificial intestinal fluid for the same time. Regarding stability in artificial gastric fluid, the MIC of C-LR18 increased by 8 times and ≥32 times after treatment with artificial gastric fluid for 30 min and 60 min, respectively, while the MIC of LR18 increased by 16 times and ≥64 times, respectively, after treatment with artificial gastric fluid for the same time. The results indicated that the stability of C-LR18 was improved both in artificial intestinal fluid and artificial gastric fluid, and the stability of C-LR18 was higher than that in artificial gastric fluid.

The impact of different salts, pH, and temperature on C-LR18 antimicrobial activity was tested by determining the effect on their MIC. The MIC of LR18 and C-LR18 to *E. coli* ATCC25922 in different physiological salt solutions, pHs, and temperatures are shown in Table 4. All univalent (Na^+^, K^+^, and NH^4+^) ions and bivalent (Mg^2+^, Zn^2+^ and Ca^2+^) and trivalent (Fe^3+^) salt ions had little effect on the antibacterial activity of LR18 and C-LR18, indicating that cyclic peptide C-LR18, like linear peptide LR18, showed good physiological salt tolerance. In addition, C-LR18 also had good thermal stability and pH stability, like linear peptide LR18. After treatment at 100 °C for 30 min, acid treatment (pH4) for 1 h, and alkali treatment (pH10) for 1 h, its MIC to *E. coli* ATCC25922 was only expanded by 2 times.

### 2.7. The Half-Life of Cyclic Peptide C-LR18 Was Prolonged

**Figure 4:**
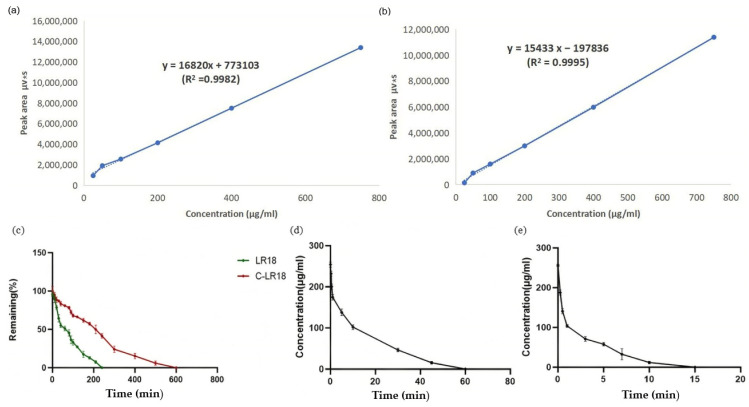
The half-life determination results of peptide C-LR18 in plasma and in SD rats. (**a**) The standard curve of LR18; (**b**) the standard curve of the C-LR18; (**c**) residual percentage of LR18 and C-LR18 in vitro plasma; (**d**) the average blood drug concentration–time curve after iv C-LR18; (**e**) the average blood drug concentration–time curve after iv LR18.

The standard curves of LR18 and C-LR18 in SD rat plasma are shown in Figure 4a,b, and the corresponding linear regression equations are Y = 16820X + 773103 (R^2^ = 0.9982 > 0.99) and Y = 15433X − 197836 (R^2^ = 0.9995 > 0.99), respectively. Based on the standard curve, the linear range of peptides LR18 and C-LR18 in rats’ plasma was (25–700) μg/mL.

To determine the half-life of the peptide in rat plasma in vitro, C-LR18 and LR18 were incubated in rat plasma for different times and then determined using HPLC. The measured peak area is substituted into the above standard curve to calculate the peptide concentration at different time points. The graph is drawn with different sampling times (min) as the horizontal coordinate and the remaining percentage (%) of LR18 and C-LR18 as the vertical coordinate (Figure 4c). The longer the interaction time of peptide with plasma, the lower the peptide content, indicating that the antimicrobial peptides were continuously degraded by protease in plasma. More than half of C-LR18 was degraded in plasma after 210 min, and no C-LR18 could be detected in plasma samples after 600 min. The half-lives of C-LR18 and LR18 in rat plasma were 209.45 ± 20.21 min and 62.12 ± 5.66 min, respectively. It can be seen that the half-life of the cyclic peptide C-LR18 in rat plasma was prolonged by 147.33 min, which was 3.37 times that of the original peptide.

To determine the half-life of the peptide in rats, SD rats were injected with antimicrobial peptides C-LR18 and LR18 through the tail vein, and blood samples were collected at different time periods and determined using HPLC. With time as the abscissa and the concentration of peptide in blood as the ordinate, the measured data were imported into GraphPad Prism 8 software for drug-time curve fitting. Figure 4d,e show the drug–time curves of LR18 and C-LR18 in rats, respectively. The half-lives of C-LR18 and LR18 in rats are shown in Table 5; they are 12.73 ± 0.79 min and 2.73 ± 0.74 min, respectively. It can be seen that the half-life of the cyclic peptide C-LR18 in rats was prolonged by 10 min, which was 4.46 times that of the original peptide. The plasma pharmacokinetic parameters of rats after caudal intravenous administration of LR18 and C-LR18 are shown in Table 5.

### 2.8. C-LR18 Has Therapeutic Effect on E. coli-Infected Mice

Antimicrobial peptide C-LR18 has low toxicity to mice. The injection dose and survival of the antimicrobial peptide C-LR18 are shown in Table 6. According to the data in the table, the reasonable dose range for detecting the LD50 of antimicrobial peptide C-LR18 in mice was determined to be 30–75 mg/kg. The physiological state of KM mice in each group was changed after different doses of antimicrobial peptide C-LR18 were injected into the tail vein, and the mice showed symptoms such as lethargy and a decreased intake of food and water. The symptoms of the mice that did not die after challenge were relieved the next day. The dosage of antimicrobial peptide C-LR18 administered via the tail vein and the survival of mice are shown in Table 6. According to the modified Kohl’s method, the LD50 of antimicrobial peptide C-LR18 injected through the tail vein in Kunming mice was 37.8 mg/kg, and its 95% confidence limit was 28.0–50.1 mg/kg.

**Figure 5:**
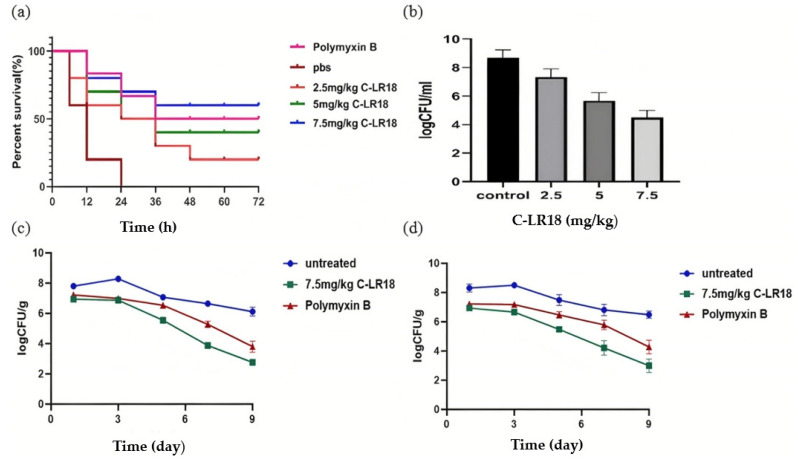
The therapeutic effect of C-LR18 on *E. coli*-infected mice. (**a**) Survival rate of *E. coli*-infected mice after treatment with different dose of C-LR18; (**b**) the number of peritoneal colonies in *E. coli*-infected mice after treatment with C-LR18; (**c**) changes of bacterial colonization in liver of *E. coli* infected mice after treatment with C-LR18; (**d**) changes of bacterial colonization in spleen of *E. coli*-infected mice after treatment with C-LR18.

Antimicrobial peptide C-LR18 has a good therapeutic effect on *E. coli*-infected mice. One hour after mice were intraperitoneally injected with 0.5 mL of *E. coli* Y1 suspension (10 9 CFU/mL), different doses of C-LR18 (2.5 mg/kg, 5 mg/kg, and 7.5 mg/kg) and polymyxin B (5 mg/kg) were intraperitoneally injected into mice. The results are shown in Figure 5a within 72 h; all mice in the PBS control group died within 24 h. The survival rate of mice in the high- (7.5 mg/kg), middle- (5 mg/kg), and low (2.5 mg/kg)-dose groups of C-LR18 was 60%, 40%, and 20%, respectively, which indicated that antimicrobial peptide C-LR18 has a good therapeutic effect on *E. coli*-infected mice. The results of the bacterial colony count in the mouse abdominal cavity showed that the number of colonies in the control group was 1 × 10^9^ CFU/mL, while the number of colonies (Figure 5b) in the 7.5 mg/kg, 5.0 mg/kg, and 2.5 mg/kg C-LR18-treated groups was reduced to 10^4^ CFU/mL, 10^6^ CFU/mL, and 10^7^ CFU/mL, respectively. It can be seen that the antibacterial peptide C-LR18 has a certain killing effect on *E. coli* infection in mice.

C-LR18 has a certain therapeutic effect on organ infection caused by *E. coli* infection in mice. The measurement results of bacterial load in liver and spleen of mice are shown in Figure 5c,d. At 72 h after *E. coli* infection, the bacterial colonization in the negative control group reached the peak and then decreased slightly for several days. There was bacterial colonization in the liver and spleen of mice in the C-LR18 treatment group and the polymyxin B control group, but the amount of bacterial colonization was significantly less than that in the negative control group. With the extension of time, the bacteria carrying capacity in liver and spleen of mice in the C-LR18 treatment group decreased significantly, and the bacteria carrying capacity was less than that in the polymyxin B group. It can be seen that C-LR18 has a certain therapeutic effect on organ infection caused by *E. coli* infection in mice.

## 3. Discussion

Antimicrobial peptides have attracted much attention from researchers for many years due to their broad spectrum and high antibacterial activity in vitro. More than 3000 AMPs have been found so far. However, only a few AMPs have been used as therapeutic agents. Poor stability, short half-life, and easy removal by enzymes in plasma are the main reasons that hinder their clinical application as therapeutic agents. Therefore, improving the stability and prolonging the half-life of antimicrobial peptides have become the focus of the research and development of new antimicrobial peptide preparations. Cyclization of linear peptides is a modification method to improve the stability of peptides, prolong their half-life, and increase the bioavailability of peptides. Compared with linear peptides, the side chains of cyclic peptides are more tightly arranged, which makes the peptide conformation more stable. Due to the lack of amino and carboxyl termini, cyclic peptides are more resistant to peptidases and endopeptidases, thus showing better in vivo stability. Gunasekera S et al. [17] synthesized skeletally looped KR-12 dimers and studied their antimicrobial activity and proteolytic stability. Their study showed that dimerization and main chain looping were effective strategies to improve the antimicrobial activity and stability of linear antimicrobial peptides. Lee CH et al. [18] used the coupling reagent DMTMM to generate succinimide in LL37-derived peptide KR12 for cyclizing, and compared with unmodified KR12, the serum stability of KR12 containing succinimide increased. Etayash et al. [5] used head–tail cyclization, side chain and tail cyclization, and added two cysteines to form disulfide bonds to cyclize IDR 1018, respectively, and found that the three cyclic peptides had a tolerance time of 120 min to trypsin, while the template peptide was completely degraded in less than 30 min. In this study, cysteine was added to the N-terminal and C-terminal of linear peptide LR18, and a new cycloantimicrobial peptide, named C-LR18, was designed and synthesized by means of end-to-end cyclization via disulfide bonds. The stability of cyclic peptide C-LR18 in protease was significantly improved. The antibacterial activity of C-LR18 against *E. coli* ATCC25922 remained unchanged after treatment with 1 mg/mL papain for 1 h, while that of the original peptide LR18 was reduced by at least 54 times. The half-life of cyclic peptide C-LR18 in rat plasma was prolonged by 147.33 min, which was 3.37 times that of the original peptide. And the half-life of C-LR18 in SD rats was also prolonged by 4.66 times that of the original peptide. Our results indicated that cyclization modification helped improve the anti-enzymatic hydrolysis ability and enhance the stability of the peptide.

Antimicrobial peptides have a variety of ways to play their role in the body. Intravenous or subcutaneous drug delivery has low requirements for the stability of the peptide itself, but oral administration undoubtedly exposes antimicrobial peptides to proteases secreted by serum, the digestive tract, and even bacteria [23]. In order to evaluate the possibility of the oral administration of antimicrobial peptide C-LR18, artificial gastric fluid and artificial intestinal fluid were used to simulate the gastrointestinal environment of animals in this study, and the MIC of C-LR18 to *E. coli* ATCC25922 was determined after treatment with artificial gastric fluid and artificial intestinal fluid. The MIC of C-LR18 increased by 4 times and 16 times after treatment with artificial intestinal fluid for 30 min and 60 min, respectively. But the MIC increased by 8 times and 32 times after treatment with artificial gastric fluid for 30 min and 60 min, respectively. These results indicated that C-LR18 is more stable in the intestine than in the stomach. It is suggested that C-LR18 can be encapsulated into an oral preparation using a non-toxic material that is stable to the gastric environment but sensitive to the intestinal environment, but this idea needs to be further verified by experiments.

An in vivo toxicity test is an essential step in the safety evaluation of exogenous drugs. The purpose is to observe the systemic toxicity of drugs on animals, including whether there are adverse physiological reactions, pathological changes in organs and tissues of the whole body, and the mortality of animals. Therefore, before the treatment of C-LR18 on *E. coli*-infected mice, we tested the acute toxicity of C-LR18 in mice. The LD50 of C-LR18 in mice was 37.8 mg/kg after a single injection of drug into the tail vein. This toxicity is significantly lower than that of many antimicrobial peptides reported so far, such as melittin (LD50 = 4.98 mg/kg) [24] and polymyxin B (LD50 = 6.52 mg/kg) [25], which was consistent with the results of in vitro hemolysis and cytotoxicity tests of C-LR18. The result of the systemic toxicity test provided a dose reference for the pharmacodynamic study of antimicrobial peptide C-LR18 in animals.

The in vitro antibacterial activity study showed that C-LR18 had a strong inhibitory effect on a variety of Gram-positive and Gram-negative bacteria. We expected that C-LR18 would also have high antibacterial activity in animals, but the in vivo environment is more complex than in vitro, and the activity and stability of antimicrobial peptides are affected by PH, salt ionizers, serum, and various proteases in vivo. Therefore, in this study, a mouse model of E. coli infection was constructed, and then the mice were treated with different doses of C-LR18. The therapeutic effect of C-LR18 was evaluated by measuring the survival rate of the mice, the number of colonies in the abdominal cavity, and the bacterial load in the liver and spleen. The results showed that the survival rate of mice treated with the 7.5 mg/kg C-LR18 group was 60%, slightly higher than those treated with 5.0 mg/kg of polymyxin B (50%). The number of colonies in the abdominal cavity of mice treated with 7.5 mg/kg C-LR18 was reduced to 10^4^ CFU/mL, which was significantly lower than that in the control group (1 × 10^9^ CFU/mL). The bacteria carrying capacity in the liver and spleen of mice in the C-LR18 treatment group decreased significantly, which was less than that in the polymyxin B group. These results indicated that C-LR18 had a therapeutic effect on *E. coli*-infected mice.

## 4. Materials and Methods

### 4.1. Materials

*E. coli* K88, *E. coli* ATCC 25922, *Staphylococcus aureus* ATCC 25923, *Staphylococcus aureus* ATCC 29213, *Klebsiella pneumoniae* CMCC 46117, *Streptococcus faecalis* ATCC 29212, and *RAW264.7* cells were obtained from the pharmacology and toxicology laboratory of the College of Veterinary Medicine, Jilin Agricultural University (Changchun, China). TransDetect Cell Counting Kit-8 (CCK-8) and Dulbecco’s modified Eagle’s medium (DMEM) were purchased from TransGen Biotech (Beijing, China). Fetal bovine serum (FBS), fetal bovine serum, papain, trypsin, pepsin, carboxypeptidase, Triton X-100, Trifluoroethanol (TFE), and phosphate-buffered saline (PBS) solution were purchased from Solarbio (Beijing, China). Artificial gastric fluid and artificial intestinal fluid were purchased from Saint-bio (Shanghai, China). Mueller-Hinton broth (MHB), Mueller-Hinton agar (MHA), and bovine serum albumin (BSA) were purchased from GL Biochem (Shanghai, China). Specific Pathogen Free (SPF) SD rats (200 ± 20 g) were purchased from Liaoning Changsheng Biotechnology Co., Ltd. (Shenyang, China). Specific Pathogen Free (SPF) KM mice were purchased from the Laboratory Animal Center of Jilin University, (Changchun, China) The animals were maintained at constant room temperature [(25 ± 2) °C] with free access to food and water for 24 h or more before experiments. All animal experiments comply with the 3R principle and comply with animal ethics standards. The ethical review board reference number is 20210610001, and the approval date is 18 June 2021.

### 4.2. Design, Synthesis and Sequence Analysis of Cyclic Peptide C-LR18

The cyclic peptide C-LR18 and the original peptide LR18 were synthesized and purified by GL Biochem (Shanghai, China). The purity of peptides was over 98%, determined using high-performance liquid chromatography purification. The molecular mass of peptides was analyzed using LC-MS.

Peptide structure drawing software: MarvinSketchV20.8, ChemDraw3DV16.0, MercuryV6.0.0.

Physical and chemical parameters of peptides were analyzed online: http://web.expasy.org/ (accessed on 21 March 2021).

### 4.3. CD Spectroscopy of Cyclic Peptide C-LR18

The CD Spectroscopy of cyclic peptide C-LR18 was detected on a Bio-Logic MOS-500 CD spectropolarimeter (Bio-Logic, Seyssinet-Pariset, France) at 25 °C according to the method described in [22]. The final concentration of C-LR18 in 10 mM PBS (pH 7.4), 30 mM SDS, and 50% TFE was 150 µM. The test conditions were as follows: wavelength range of 190–250 nm, speed of 10 nm/min, and light diameter of 1 nm. C-LR18 was scanned three times and averaged to generate the final spectra. Circular dichroism data were analyzed according to the following formula to obtain the mean residue ellipticity (θM) (deg·cm^2^·dmol^−1^):

(1) $$\theta M=\frac{\theta obs\times 1000}{1\times c\times n}$$

In the formula, θobs represents the measured ellipticity (mdeg) obtained by scanning in the wavelength range, l represents the light diameter length (cm), c represents the peptide concentration (mM), and n represents the amino acid number of the peptide C-LR18.

### 4.4. Antimicrobial Activity Assay

#### 4.4.1. Minimum Bacteriostatic Concentration (MIC) Determination

The MIC test was performed using the method recommended by the National Committee for Clinical Laboratory Standards (NCCLS) Standards and Guidelines, and the method was modified according to the characteristics of antimicrobial peptides [26]. The bacteria were cultured at 37 °C for 16 h, and the microbial suspension was diluted to a final concentration of 5 × 10^5^ CFU/mL. Then, 50 µL of bacterial solution was mixed with 50 µL of MHB containing different concentrations peptides (0.5–256 µM) in a 96-well plate. After incubation at 37 °C for 16 h, MIC was examined by measuring the OD value at 492 nm (Microplate reader, TECAN GENios F129004, Tecan, Salzburg, Austria). The assays were performed three times. Geometric mean MIC represents the average MIC of the peptide against all the tested strains.

#### 4.4.2. Minimum Bactericidal Concentration (MBC) Determination

MBC detection was performed according to the method of Taiyari et al. [27]. According to the MIC results, 50 µL of bacterial cultures was absorbed from the minimum inhibitory concentration hole and the three previous holes, respectively; transferred to a new MH solid medium; and incubated in an incubator at 37 °C overnight, and the bacteria on the plate were counted. The concentration of antibacterial peptide that kills 99.99% of the tested bacteria is defined as the minimum bactericidal concentration (MBC) of the peptide. The experiment was repeated three times. Geometric mean MBC represents the average MIC of the peptide against all the tested strains. Therapeutic index (TI) represents the ratio of the minimum concentration of peptide that causes hemolysis of 10% of erythrocytes to the GM MIC (added in the Methods section).

#### 4.4.3. Bactericidal Kinetics Assay

Bactericidal kinetics assay was performed according to the method outlined by Zhang et al. [28]. *E. coli* ATCC25922 of 1 × 10^6^ CFU/mL was mixed with MHB containing different concentrations of AMPs; the final concentrations of AMPs were set to 1 × MIC and 2 × MIC, respectively. The mixed solutions were incubated in a shaker at 37 °C. A total of 100 μL of the above mixed solution was absorbed every 20 min and diluted with sterile MH liquid medium, and then 50 μL of bacterial solution was aspirated and evenly coated on the solid MH plate. After incubation at 37 °C for 16–18 h, the bacteria colonies on the plate were counted. The bactericidal kinetics curves of LR18 and C-LR18 against *E. coli* ATCC25922 were plotted. The experiment was repeated three times.

### 4.5. Antimicrobial Mechanism Study

#### 4.5.1. Membrane Permeability Assay

The effect of C-LR18 on bacterial membrane permeability was measured using the propidium iodide (PI) uptake assay described in [22]. *E. coli* ATCC25922 in logarithmic growth phase were adjusted to 1 × 10^6^ CFU/mL. C-LR18 was added with the final concentration of 4 μM. The mixtures were incubated for 30 min at 37 °C. Then, PI was added at a concentration of 10 mg/mL, incubated for 10 min at room temperature in the dark, and compared with the bacteria treated with PBS (negative control) and bacteria treated with melittin (25 μg/mL) (positive control). After eliminating unbound dye by washing twice with PBS, the bacterial cells were detected using a flow cytometer (Ex = 535 nm/Em = 615 nm). The results were analyzed using FlowJo 6.2.1 and GraphPad Prism 5 software.

#### 4.5.2. Scanning Electron Microscopy

*E. coli* ATCC25922 cells were washed three times in 10 mM PBS, diluted to 10^7^ CFU/mL, and treated with 4 μg/mL C-LR18, and bacteria without peptide treatment were used as negative controls. After incubation at 37 °C for 1 h and centrifuging at 3000 r/min for 10 min, the super serum was discarded. Then, 2.5% glutaraldehyde solution was added to fix for 6 h. After fixation, it was washed with PBS for 2–3 times; washed with 30%, 50%, 70%, and 90% ethanol solution; and 100% ethanol dehydration. It was then scanned using an Extreme-resolution Analytical Field Emission SEM (Mira 3 XH, Tescan, Brno, Czech) after coating with gold–palladium.

#### 4.5.3. DNA Binding Assay

The DNA binding test was performed according to gel retardation experiments described previously [22]. Firstly, genomic DNA was extracted from *E. coli* ATCC25922 using a DNA extraction kit. A total of 100 ng of genomic DNA was mixed with 1–512 µM peptide in a binding buffer (50 µg/mL of BSA, 1 mM of ethylene diamine tetra-acetic acid, 20 mM of KCl, 10 mM of Tris-HCl (pH = 8.0), 5% glycerol, and 1 mM of dithiothreitol) and incubated at 37 °C for 60 min. Subsequently, the samples were examined using 0.8% agarose gel electrophoresis. Observation and photography were performed in the UVP gel imaging system, and the electrophoresis mobility was analyzed.

### 4.6. Hemolysis Activity Determination

The hemolytic activity was examined according to the literature [29]. Briefly, the collected rabbit red blood cells [purchased from Oumarsi (Shanghai, China) Biotechnology Co., Ltd.] were washed three times with PBS (pH 7.4) and diluted to 2% (*v*/*v*). A total of 100 µL of rabbit red blood cells suspension were mixed with 100 µL of PBS (pH 7.4) containing different concentrations of peptides (1–256 µM) in a 96-well plate. The positive control was a mixture of 100 µL of red blood cell suspension and 100 µL of 0.2% TritonX-100 solution, and the negative control was a mixture of 100 µL of red blood cell suspension and 100 µL of PBS solution. The mixtures were incubated at 37 °C for 1 h, then centrifuged at 1000× *g* for 10 min. The OD value at 570 nm of the supernatant was measured using a microplate reader (TECAN GENios F129004, Tecan, Salzburg, Austria). The hemolytic index was calculated according to the following formula:

Hemolytic index % = (OD peptide − ODPBS)/(ODT ritonX-100 − ODPBS) × 100%(2)

The concentration of the antimicrobial peptide causing 10% erythrocyte hemolysis was defined as the minimum hemolytic concentration of the antimicrobial peptide on rabbit red blood cells. The experiment was repeated three times.

### 4.7. Cytotoxicity Determination

The cytotoxicity of LR18 and C-LR18 to eukaryotic *RAW264.7* was determined using CCK-8 assay described by Jia et al. [26]. Briefly, RAW264.7 cells were placed into 96-well plates at a density of 2.0 × 10^4^ and then cultured at 37 °C under conditions of 5% CO_2_ for 12–16 h. After the cells adhered to the wall, 50 μL of different concentrations (0.5–256 µM) of AMPs in DMEM were added to Wells 1–10. A 100 μL cell suspension was added to well 11 as positive control, and 100 μL of DMEM was added to well 12 as a negative control. The 96-well plates were cultured in 37 °C 5% CO_2_ incubator for 12–16 h; then, CCK-8 (10%, *v*/*v*) was put into each well and incubated at 37 °C for 4 h. The cytotoxicity assay was examined by measuring the OD value of the mixtures at 450 nm. The assays were performed three times.

### 4.8. Stability Assays

#### 4.8.1. Impact of Salts, pH, and Temperature on Antimicrobial Activity

The impact of salts, pH, and temperature on antimicrobial activity of peptides was determined according to the literature [26]. The impact of different salts on peptides’ antimicrobial activity was tested by determining the effect on their MIC. Peptides were diluted in the MHB medium containing 2.5 mM of CaCl_2_, 150 mM of NaCl, 4.5 mM of KCl, 6 mM of NH_4_Cl, 1 mM of MgCl_2_, 8 mM of ZnCl_2_, and 4 mM of FeCl_3_, respectively. Then, the MIC of peptides treated with different salts for *E. coli* ATCC 25922 was detected; subsequent steps were the same as for the MIC assay.

The impact of temperature on peptides’ antimicrobial activity was also tested. Peptides were incubated at 0 °C, 37 °C, and 100 °C for 30 min, respectively, and then the MIC of peptides to *E. coli* ATCC 25922 was detected; subsequent steps were the same as for the MIC assay.

To detect the impact of pH on peptides’ antimicrobial activity, peptides were diluted in different pH (4.0, 6.0, 8.0, and 10.0) solutions and cultured for 1 h, and then the MIC of peptides to *E. coli* ATCC 25922 was detected; subsequent steps were the same as for the MIC assay.

#### 4.8.2. The Stability of Peptide in Protease, Serum, and GastroIntestinal Conditions

The stability of LR18 and C-LR18 in protease, serum, and gastrointestinal conditions was determined using MIC assay, as described previously [19,26].

To detect the stability of peptides in protease, the peptides were diluted in 1 mg/mL of trypsin, pepsin, papain, and protease K, respectively, and incubated at 37 °C for 1 h. Then, the protease in the peptide solutions was inactivated in a water bath at 60 °C for 30 min. Subsequent steps were the same as for the MIC assay. The assays were performed three times.

To detect the stability of peptides in serum, the peptides were mixed with various concentrations of fetal bovine serum and mouse serum solutions, and the final concentrations of serum were 6.25%, 12.5%, 25%, and 50%. The MIC of peptides against *E. coli* ATCC25922 in the presence of different concentrations of serum was determined. Subsequent steps were the same as for the MIC assay. The assays were performed three times.

The stability in gastrointestinal conditions was also determined. The peptides were diluted in artificial gastric fluid and artificial intestinal fluid, digested in water bath at 37 °C for 0.5 and 1 h, and then inactivated in water bath at 60 °C for 30 min. Then, the PH of the solution was adjusted to 7.0 with 10% Na_2_CO_3_. Subsequent steps were the same as for the MIC assay. The assays were performed three times.

### 4.9. Half-Life Determination

#### 4.9.1. Half-Life Determination of Peptides in Plasma In Vitro

Half-life of peptides in vitro was determined using HPLC according to the method described in [30]. Blood from SPF SD rats was collected in a centrifuge tube containing 0.5% heparin sodium via cardiac sampling. Plasma was separated and stored at −80 °C until analyzed. The plasma samples with final concentrations of 25 μg/mL, 50 μg/mL, 100 μg/mL, 200 μg/mL, 400 μg/mL, and 750 μg/mL were prepared by mixing 1500 μg/mL of antimicrobial peptides with rat plasma, then adding acetonitrile containing 0.1% TFA at 1/1 volume. After vortexing for 30 s, the samples were centrifuged at 14,000 r/min for 20 min, the supernatant was aspirated, sterilized using a 0.22 μm sterilization filter, and 30 μL of the sample was used for HPLC determination. After HPLC detection, the value of the chromatographic peak area was counted, the mathematical relationship between the sample concentration and the chromatographic peak area was calculated, and the standard curve was drawn. The standard curve was regressio-calculated by linear fitting Y = aX + b and weighted least square method (w = 1/c2).

The 1 mg/mL peptide solution was mixed with SD rat plasma and incubated in a water bath at 37 °C. Samples were taken at different time points of 0, 20, 30, 40, 60, 80, 100, 120, 140, 160, 180, 200, 210, 240, 300, 400, 500, and 600 min. After vortexing for 30 s, the samples were centrifuged at 14,000 r/min for 20 min, the supernatant was aspirated and sterilized using a 0.22 μm sterilization filter, and 30 μL of the sample was used for HPLC determination. The experiment was repeated three times. The peak area measured by HPLC was substituted into the standard curve obtained by the above experiment, the peptide concentration at different time points was calculated, and the half-life of peptides in plasma was calculated.

#### 4.9.2. Half-Life Determination of Peptides in SD Rats

Half-life of peptides in SD rats was determined via HPLC according to the method described in [31]. The SD rats were maintained at constant room temperature [(25 ± 2) °C] with free access to food and water for 48 h. Each SD rat was weighed to an accuracy of 0.10 g, numbered, and grouped randomly, with 6 rats in each group. LR18 and C-LR18 were injected into SD rats via tail vein at a dose of 5 mg/kg body weight. A total of 200 μL of blood was collected from the orbital venous plexus at different time points of 0, 0.5, 0.8, 1.0, 1.5, 2.5, 3.5, 5, 10, 30, and 60 min, and placed into a centrifuge tube containing 0.5% heparin sodium. After centrifugation at 3000 r/min for 15 min, the supernatant was absorbed into a new centrifuge tube, and then acetonitrile containing 0.1% TFA was added at 1/1 volume. Vortexing for 30 s, the samples were centrifuged at 14,000 r/min for 20 min, the supernatant was aspirated and sterilized using a 0.22 μm sterilization filter, and 30 μL of the sample was used for HPLC determination. The pharmacokinetic parameters were calculated using Phoenix Winnonlin 7.0 software and analyzed with non-atrioventricular model. Drug–time curves were plotted using Graphpad Prim, and Mean ± SD was used to interpolate each point during drug–time curve fitting.

### 4.10. Therapeutic Experiments in Mouse Models Infected with E. coli

#### 4.10.1. Acute Toxicity of Antimicrobial Peptide C-LR18 in Mice

To obtain the reasonable dose range of C-LR18 on mouse LD50, KM mice (18–21 g) were randomly divided into 5 groups with 8 mice in each group. Different doses of C-LR18 (100 mg/kg, 75 mg/kg, 50 mg/kg, 30 mg/kg, and 10 mg/kg) were injected into the tail vein of mice in different groups. The health status of the mice was observed, dose data leading to 100% (10/10) and 0% (0/10) death of the mice was recorded, and the reasonable dose range of C-LR18 on mouse LD50 was determined.

To determine the median lethal dose (LD50) of C-LR18 in mice, KM mice (18–21 g) were randomly divided into 5 groups with 10 mice in each group; different doses of C-LR18 (77 mg/kg, 64 mg/kg, 53 mg/kg, 44 mg/kg, and 36 mg/kg) were injected into the tail vein of mice in different groups. The health status of mice was observed, and the mortality data of 5 groups of mice were recorded. The LD50 and 95% confidence limit of C-LR18 for mice were calculated according to the following formula:

LD50 = log − 1[*Xm* − i(Σ*P* − 0.5)] (3)

(4) $$\mathrm{SX}50=i\sqrt{\sum \frac{pq}{n}}$$

95% Confidence limits of LD50 = log − 1(log − 1LD50 ± 1.96SX50)(5)

*Xm*: dose pair value of maximum dose group, i: the difference between the dose pairs of two adjacent groups, *P*: animal mortality in each group, Σ*P*: the sum of the mortality of each group of animals, *n*: number of animals per group, *q*: survival rate per dose group, *q* = *p* − 1.

SX50: Standard error of logLD50.

#### 4.10.2. Establishment of a Mouse Model Infected with *E. coli*

The mice (female, 18–21 g) were randomly divided into 6 groups, with PBS dilution as the control group. A total of 1–5 groups were injected intraperitoneally with 0.5 mL of *E. coli* solution containing 1010, 109, 108, 107, and 106 CFU/mL, respectively. After inoculation, the mice were observed continuously for 5 days, and the mortality rate was recorded. The minimum dose (LD 100) at which all mice die within 5 days was used as the optimal dose for the bacterial attack in this experiment.

#### 4.10.3. Therapeutic Experiments in Mouse Models

In order to further evaluate the therapeutic potential of antimicrobial peptide C-LR18, a mouse model infected with *E. coli* was constructed in this study. The Km mice (female, 18–21 g) were maintained at constant room temperature [(25 ± 2) °C] with free access to food and water for 48 h. Each Km mouse was weighed to an accuracy of 0.10 g and numbered. According to the principles of safe pharmacology research (generally no less than 10 small animals per group and no less than 6 large animals per group), Km mice were randomly divided into 5 groups with 10 mice in each group. A total of 0.5 mL of *E. coli* Y1 suspension (10^9^ CFU/m L) was intraperitoneally injected into each group of mice. One hour after challenge, different doses of C-LR18 (2.5 mg/kg, 5 mg/kg, and 7.5 mg/kg) and polymyxin B (5 mg/kg) were intraperitoneally injected into mice. The group injected with the same dose of physiological water was the control group. The health status of the mice was observed, and the mice that did not reach the criteria for euthanasia within 48 h were defined as “survival” in this experiment.

#### 4.10.4. Determination of Bacterial Colony Number in Mouse Abdominal Cavity

One hour after the mice were intraperitoneally injected with 0.5 mL of *E. coli* Y1 suspension (10^9^ CFU/mL), different doses of C-LR18 (2.5 mg/kg, 5 mg/kg, and 7.5 mg/kg) were injected into the mice through the tail vein, respectively. After 24 h, 2 mL sterile saline was injected into the abdominal cavity of the mice, and the mice were killed via cervical dislocation. The peritoneal cavity of mice was opened, the peritoneal fluid was absorbed, and the peritoneal fluid was diluted with sterilized MH medium in a double ratio. After coating the plate, the peritoneal fluid was cultured at 37 °C for 16–20 h, and the colony count was performed. Three mice were taken from each group.

#### 4.10.5. Determination of Bacterial Load in Liver and Spleen of Mice

One hour after the mice were intraperitoneally injected with 0.5 mL of *E. coli* Y1 suspension (10^9^ CFU/mL), C-LR18 was injected into the mice at a dose of 7.5 mg/kg through the tail vein. The normal saline group was used as negative control, and the polymyxin B group (1 mg/kg) was used as positive control. After that, one mouse was taken every 24 h, sacrificed via cervical dislocation, and the liver and spleen were removed, weighed, and added to PBS buffer for grinding at 3000 r/min for 2 min. The abrasive solution was diluted with sterile PBS (1:10,000), and 0.1 mL diluent was evenly coated in solid MH medium and cultured at 37 °C overnight. Colony counting was performed on the samples, and the bacterial colonization amount in liver and spleen was calculated.

## 5. Conclusions

In this study, a new cyclic antimicrobial peptide, named C-LR18, was designed and synthesized. C-LR18 retained the advantages of low hemolysis and low cytotoxicity of the original peptide. C-LR18 had higher antibacterial activity and stability than the original peptide, and the half-life is also longer in rats both in vivo and in vitro Moreover, C-LR18 had a therapeutic effect on *E. coli*-infected mice and is expected to be developed into a therapeutic drug for bacterial diseases and applied to the treatment of human and veterinary diseases.

## Figures and Tables

**Table 1:** Physicochemical parameter analysis of peptides.

Peptides	Sequence	Molecular Mass	Isoelectric Point	Net Charge	Hydrophobicity	Instability Index
LR18	LRRLLRLPRRPLRLLRRL-NH2	2367.02	12.85	8	0.489	164.92
C-LR18	CLRRLLRLPRRPLRLLRRLC	2573.3	12.22	8	0.19	159

**Table 2:** MICs (MBCs), geometric mean MIC (MBC), and therapeutic index (TI) of peptides against bacteria (μM).

	C-LR18	LR18	Melittin
MIC (MBC) (μM)			
*E. coli* ATCC25922	4(8)	2(4)	1(2)
*E. coli* K88	1(2)	2(4)	2(4)
*K. pneumonia* CMCC46117	1(2)	4(4)	4(8)
*S. aureus* ATCC29213	2(4)	8(16)	1(2)
*S. aureus* ATCC25923	8(16)	4(8)	2(4)
*E. faecalis* ATCC29212	16(32)	16(32)	2(4)
Geometric mean MIC (MBC) (μM)			
Gram-negative bacteria	2(4)	2.7(4)	2.33(4.67)
Gram-positive bacteria	8.7(17.3)	9.3(18.7)	1.67(3.33)
MHC10	128	128	1
Therapeutic index (TI)			
Gram-negative bacteria	64	47.4	0.43
Gram-positive bacteria	14.71	13.76	0.6

**Table 3:** MIC of peptides treated with protease, serum, and artificial gastric and artificial intestinal fluids against *E. coli* ATCC25922 (μM).

		**Protease (1 mg/mL)**							
	Blank Control	Trypsin		Pepsin		Papain	Protease K	Carboxypeptidase	
LR18	2	2		>128		>128	>128	>128	
C-LR18	4	4		>128		4	>128	8	
		**Artificial Gastric Fluid**				**Artificial Intestinal Fluid**			
	Blank control	30 min		60 min		30 min		60 min	
LR18	2	32		>128		16		64	
C-LR18	4	32		>128		16		64	
		**Mouse Serum**				**Fetal Bovine Serum**			
	Blank control	6.25%	12.50%	25%	50%	6.25%	12.50%	25%	50%
LR18	2	4	8	16	32	8	8	16	32
C-LR18	4	8	8	16	16	8	8	16	16

**Table 4:** MIC of peptides treated with physiological salt solution (μM), temperature, and pH against *E. coli* ATCC25922.

**Physiological Salt Solution**								
	Blank control	CaCl_2_	NaCl	KCl	NH_4_Cl	MgCl_2_	ZnCl_2_	FeCl_3_
LR18	2	2	2	2	2	2	2	2
C-LR18	4	4	4	4	4	4	4	4
		**Temperature**			**pH**			
	Blank control	0 °C	37 °C	100 °C	pH4	pH6	pH8	pH10
LR18	2	2	2	8	4	2	2	4
C-LR18	4	4	4	8	8	4	4	8

**Table 5:** The main pharmacokinetic parameters of LR18 and C-LR18 in rats after caudal vein administration (*n* = 3).

LR18			C-LR18		
Parameter	Value	Unit	Parameter	Value	Unit
AUC0-t ^a^	619.13 ± 33.4	Μg·mL^−1^ × min	AUC0-t	3376.13 ± 37.72	μg·mL^−1^ × min
MRT0-t ^b^	3.09 ± 0.29	min	MRT0-t	13.51 ± 0.31	min
Cmax ^c^	256.04 ± 3.57	μg·mL^−1^	Cmax	254.23 ± 6.30	μg·mL^−1^
T1/2β ^d^	2.73 ± 0.74	min	T1/2β	12.73 ± 0.79	min
CL ^e^	0.075 ± 0.006	min/(mL × kg)	CL	0.013 ± 0.002	min/(mL × kg)

^a^ The area enclosed by the blood concentration curve to the time axis. ^b^ The average residence time of drug molecules in the body represents the time required to eliminate 63.2% of the drug from the body. ^c^ The highest blood concentration that occurs after administration. ^d^ Terminal elimination half-life, the time required for the terminal phase blood concentration to fall by half. ^e^ The apparent volume of distribution of a drug that is cleared from the body per unit time.

**Table 6:** Results of LD50 test of antibacterial peptide C-LR18 on mice.

Group	Number of Animals	Dose (mg/kg)	Logarithm of Dose (x)	Number of Animal Deaths (r)	Mortality (p)	Survival Rate (q)
1	10	36	1.56	0	0	1
2	10	44	1.64	2	0.2	0.8
3	10	53	1.72	5	0.5	0.5
4	10	64	1.81	7	0.7	0.3
5	10	77	1.89	10	1	0

## Data Availability

The original contributions presented in this study are included in the article. Further inquiries can be directed to the corresponding authors.

## Abbreviations

The following abbreviations are used in this manuscript:

AMPs	Antimicrobial peptides.
MIC	Minimum inhibitory concentration.
MBC	Minimum bactericidal concentration.
LD50	Median lethal dose.
MHB	Mueller–Hinton broth.
